# The Obesity Paradox and Heart Failure: A Systematic Review of a Decade of Evidence

**DOI:** 10.1155/2016/9040248

**Published:** 2016-01-20

**Authors:** Emmanuel Aja Oga, Olabimpe Ruth Eseyin

**Affiliations:** ^1^Department of Epidemiology and Public Health, University of Maryland, Baltimore, MD 21201, USA; ^2^Department of Epidemiology, Harvard Chan School of Public Health, Boston, MA 02115, USA

## Abstract

There is scientific consensus that obesity increases the risk of cardiovascular diseases, including heart failure. However, among persons who already have heart failure, outcomes seem to be better in obese persons as compared with lean persons: this has been termed the* obesity paradox*, the mechanisms of which remain unclear. This study systematically reviewed the evidence of the relationship between heart failure mortality (and survival) and weight status. Search of the PubMed/MEDLINE and EMBASE databases was done according to the PRISMA protocol. The initial search identified 9879 potentially relevant papers, out of which ten studies met the inclusion criteria. One study was a randomized clinical trial and 9 were observational cohort studies: 6 prospective and 3 retrospective studies. All studies used the BMI, WC, or TSF as measure of body fatness and NYHA Classification of Heart Failure and had single outcomes, death, as study endpoint. All studies included in review were longitudinal studies. All ten studies reported improved outcomes for obese heart failure patients as compared with their normal weight counterparts; worse prognosis was demonstrated for extreme obesity (BMI > 40 kg/m^2^). The findings of this review will be of significance in informing the practice of asking obese persons with heart failure to lose weight. However, any such recommendation on weight loss must be consequent upon more conclusive evidence on the mechanisms of the obesity paradox in heart failure and exclusion of collider bias.

## 1. Introduction 

Obesity has reached epidemic proportion in many parts of the world and worldwide prevalence rates have increased rapidly since 1980 [1]. Globally, more than 1.4 billion adults aged 20 years and above were overweight in 2008, representing 35% of the world population at the time (11% were obese). In total, over 200 million men and nearly 300 million women were obese. Sixty-five (65) % of the world's population lives in countries where overweight and obesity kills more people than underweight. The epidemic is not limited to adulthood; more than 40 million children under the age of five were overweight in 2011 [2]. Obesity is a huge problem in the United States; prevalence in adults increased by 50% over 2 decades (1980s to the late 90s), with 70% of adult Americans either overweight or obese [3, 4]. Developing countries have not been spared the problem and are experiencing a high prevalence of obesity similar to trends that were experienced by developed countries in the earlier years of the obesity epidemic; this phenomenon has been tagged the “nutrition transition” [1, 5].

It is established in literature that obesity is associated with increased risk of developing cardiovascular disease, hypertension, coronary artery disease, heart failure, and stroke, and death [6–11]. It represents an epidemic with far reaching consequences on health and morbidity. However, within the last decade, mortality in obese individuals has been seen to be lower in comparison with normal weight persons [12].

## 2. The Obesity Paradox 

Longitudinal studies have shown the existence of an “obesity paradox”—a clinical phenomenon in which obese persons have a lower risk of mortality (or better survival) within clinical subpopulations [13–15]. While obesity remains associated with greater risk of mortality within the general population, among those who have already fallen ill, there are suggestions that it may offer some protection with regard to mortality [16, 17]. This paradoxical benefit of obesity has been shown to exist for a wide range of cardiovascular diseases—myocardial infarction, hypertension, patients who have had a coronary bypass, peripheral vascular disease, atrial fibrillation, and aortic stenosis, and patients with cardiac implants and other Acute Coronary Syndromes (ACS) [18–24]. This paradox also exists in patients with pneumonia, cancer, Chronic Obstructive Pulmonary Disease (COPD), renal disease, stroke, chronic respiratory insufficiency, and diabetes mellitus [25–30]. The obesity paradox has been specifically demonstrated in heart failure patients with a consistency of results seen among a wide range of clinical subpopulations across geographical locations, gender, age range, and the presence or absence of comorbidities [31], and across different measures of body fatness—Body Mass Index (BMI), Triceps Skinfold Thickness (TSF), Waist-Hip Ratio (WHR), and Waist Circumference (WC) [32–39].

*Why Heart Failure?* Excessive body weight has for long being associated with negative effects on cardiac structure, systolic and diastolic left ventricular function. Excess weight has also been known to predispose to coronary heart disease and hypertension which are major causes of heart failure. Therefore, heart failure is increasingly common in obese and overweight persons [9]. In spite of this clear evidence, obese persons with heart failure have better prognosis than their lean counterparts. The mechanisms of this obesity paradox remain a subject of fierce debate.

## 3. Rationale 

Focus is steadily shifting towards understanding the associations between body fatness and risk of disease and death for specific subpopulations rather than the general public, for which much is already known in terms of risk of disease associated with excessive weight. It has been shown with some degree of consistency that inferences about effects of excessive body fat are not consistent across all populations as now demonstrated by the obesity paradox. This systematic review is directed at understanding the effect of body fatness in patients with heart failure and at demonstrating the presence or absence of the obesity paradox in this clinical subpopulation.

## 4. Objectives

The objective of this systematic review is to assess the evidence of the relationship between heart failure mortality (or survival) and weight status.

## 5. Methods 

### 5.1. Protocol

This review was done according to Preferred Reporting Items for Systematic Reviews and Meta-Analysis (PRISMA).

### 5.2. Search Strategy and Study Selection Process

Articles were identified by search of PubMed/MEDLINE/EMBASE from the year 2004 through March 2014. Search was done between 12 (first query) and 22 April (2nd query) 2014. Focus was on longitudinal studies that documented mortality and survival in heart failure for obese versus nonobese persons (and indeed all BMI categories); articles that demonstrated the obesity paradox with respect to mortality or survival were included. Studies that reported composite outcomes (e.g., having deaths, heart transplantation, or heart device implantation as part of outcomes) rather than just deaths alone were excluded to ensure uniformity. The articles were reviewed to identify those that used standard BMI categories in longitudinal studies of heart failure mortality among adults with BMI measured or self-reported at baseline. To enable comparability across studies, those studies that reported less frequently used BMI classifications, for example, BMI quartiles, were excluded, as most studies utilize the WHO categories of BMI classification. Also, for studies that reported other measures of body composition like TSF and WC, only those that used internationally accepted categorizations were used; all others were excluded. Studies were included if measured or self-reported weight and height are recorded. Sample size, number of deaths, age at baseline, length of follow-up, hazard ratios, and 95% confidence intervals were abstracted. Studies were restricted to those published within the last 10 years. Also, only longitudinal studies were reviewed because mortality (or survival) is best studied with such designs. Finally, only studies that used the New York Heart Association (NYHA) Classification of Heart Failure were included in the review to ensure that the study population was accurately defined and homogenous across studies reviewed.

### 5.3. Search Terms

Search terms are as follows: ((((((((((((“Mortality” [Mesh]) OR “Death” [Mesh]) OR “Survival”  [Mesh]) AND “Heart Failure” [Mesh]) AND “Body Weight”  [Mesh]) OR “Obesity”  [Mesh]) OR “Body Composition” [Mesh]) OR “Body Mass Index” [Mesh]) NOT “Review” [Publication Type])) NOT “Meta-Analysis” [Publication Type]) [Mesh]).

### 5.4. Validity and Quality Assessment

Articles were evaluated based on relevance to medical practice, methodological quality, and validity of inference made. A quality assessment tool, Quality Assessment Tool for Quantitative Studies (Effective Public Health Practice Project (EPPHP)) [40], was used to grade quality of papers into strong, moderate, and weak using component and global ratings (see Table 1). The components assessed were as follows: selection bias, study design, confounders, blinding, data collection methods, withdrawals, and dropouts, intervention integrity, and analysis. Each component was graded strong, moderate, and weak, after which an overall grade was assigned to the study. Articles were first included or excluded based on title, secondly based on the abstract, and then thirdly based on a review of the full text article.

## 6. Inclusion Criteria

Inclusion criteria were as follows:

1. Studies published in a peer-reviewed journal.
2. Studies using measured or self-reported BMI, WC, WHR, and TSF.
3. Studies that must have been longitudinal studies (retrospective or prospective or clinical trial).
4. Studies that must have examined the association between body fatness and mortality/survival specifically in heart failure.
5. Study that must be a primary report (not a systematic review or meta-analysis).
6. Study that must have been published within the last 10 years.
7. Studies that must have used the New York Heart Association (NYHA) Classification of Heart Failure.

## 7. Exclusion Criteria 

Exclusion criteria were as follows:

1. Studies that use composite outcomes (e.g., studies for which outcome variable is = death or transplant or device).
2. Studies that used categorizations of measures of body fat composition, for example, Body Mass Index (BMI), Triceps Skinfold thickness (TSF), or Waist Circumference (WC) different from the WHO or another internationally comparable classification.

## 8. Results 

### 8.1. Study Selection

A total of 9,879 papers were found in the initial search. Most of these papers were dropped for several possible reasons:

1. The paper focused only on one of the core issues of interest, obesity, obesity paradox, heart failure, and mortality, and not necessarily any relationship between them.
2. The paper did not use standard categories of measures of body fat composition for exposure classification: BMI (underweight: less than 18.5 kg/m^2^; normal weight: 18.5–24.9 kg/m^2^; overweight: 25–29.9 kg/m^2^; obese: greater than 30 kg/m^2^), skin fold thickness (using reference tables for appropriate age, sex, and ethnicity of study population), Waist Circumference (high risk: women greater than 35 inches; men greater than 40 inches with concomitant BMI > 25 kg/m^2^), and Waist-Hip Ratio (high risk: men greater than 1, women greater than 0.8).
3. Studies were excluded if they were secondary studies: systematic reviews and meta-analysis; commentaries, book reviews, and “grey” literature were also excluded.

**Figure 1:**
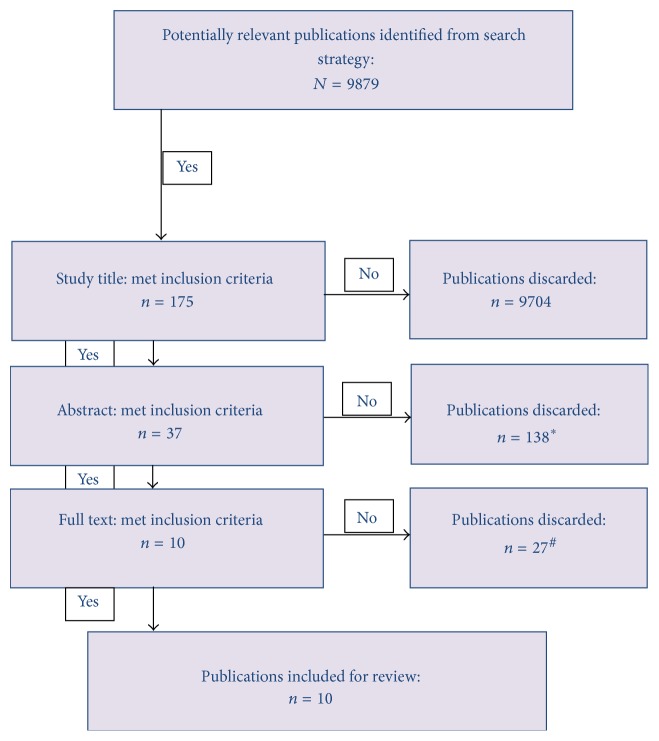
The PRISMA flowchart.  ^*∗*^Papers were secondary studies or primary studies with focus on obesity, obesity paradox, or mortality but not a relationship between them.  ^#^Papers used nonstandard categories of BMI classification and other measures of body fat composition or used composite outcome.

Overall, 10 papers met the inclusion criteria (see Figure 1).

### 8.2. Study Characteristics and Samples

Search criteria included studies published within the last 10 years, while a systematic review may cover all available studies, the most work on the obesity paradox emerged in the ten-year period covered by the review, and also studies from the previous decade will prove to be of the most benefit for decision in terms of the most recent knowledge on the subject matter. In ten studies that met criteria and were included for review, five were conducted in the US, three were in Spain, one was in Brazil, and one was in Israel. Nine were cohort studies while one was a randomized clinical trial (see Table 2). Of the studies included, average age was 63 years, with a male to female ratio of about 3 : 1 across all studies.

## 9. Discussion

The aim of this paper was to extensively review the evidence available concerning the presence of an obesity paradox in heart failure patients. This refers to the unexpectedly better prognosis of heart failure, in terms of survival and mortality, in persons who are heavier, as compared with slimmer persons as measured by the Body Mass Index (BMI) using standard categories for underweight, normal weight, overweight, and obese patients, or the Waist Circumference (WC) or Triceps Skinfold Thickness (TSF). It appears that investigators only recently began to use standard approaches in defining body fat composition or heart failure; and this explains why, despite the search criteria being tailored to find studies within the last 10 years, the only eligible studies were done in the last 5 years, a sign that researchers are beginning to standardize methods for studies in this area. It also appears that a majority of studies continue to use BMI as measure of body fat composition despite criticisms of the BMI as not being a good indicator of body fat related health outcomes [41].

All ten studies reported better prognosis (better survival, hazard ratios, or log rank) of heart failure in persons with higher BMI, WC, or TSF in comparison with the normal weight categories. The results were globally statistically significant (all categories of BMI > 25 kg/m^2^; or TSF > 20 mm; or WC > 35 inches in women and >40 inches in men) in seven studies [35, 36, 42–46]. However, despite statistically significant results for higher degrees of BMI (>30 kg/m^2^), the results were not statistically significant in the “overweight” category of BMI (25.0–29.9 kg/m^2^) in three studies [47–49]. None of the eligible studies reported a result that refuted the obesity paradox. Two studies [35, 36] reported Waist Circumference (WC) comparisons; another study reported Triceps Skinfold Thickness (TSF) [46]; still the paradox existed for two-year survival in both.

Sharma et al. conducted a meta-analysis consisting of six studies [50] with 22,807 patients having chronic HF. They investigated the relation between all-cause mortality and cardiovascular disease mortality as well as rates of hospitalization. In their findings, they reported that their analysis confirmed the existence of the obesity paradox amongst overweight and moderately obese patients with chronic HF. Over a mean follow-up period of 2.85, patients who were underweight, overweight, obese, and severely obese had RR of 1.27, 0.78, 0.79, and 0.75, respectively, for total mortality. They reported similar findings in the association between BMI and CVD mortality. However, extremely obese patients did not seem to reflect the apparent benefit of obesity in hospitalization rates with the RR for underweight, overweight, obese, and severely obese patients being 1.19, 0.92, 0.99, and 1.28, respectively [50].

The mechanism of the obesity paradox, especially in heart failure, continues to confound researchers. Overweight and obese persons often develop heart failure following hypertension and coronary heart disease due to excessive weight, usually a direct result of left ventricular dysfunction. It has been suggested that the worse prognosis in lean persons with heart failure may be due to the fact that heart failure in these persons is less frequently due to hypertension and coronary heart disease and thus possibly caused by a condition with a more devastating outcome. Also obese patients tend to present at a younger age and with less severe disease which could lead to better prognosis [9]. It must however be stated that the paradox persists even in studies that have adjusted for age at presentation and stage of disease (NYHA Classification) [42, 43, 47].

Another possible reason for these findings is that heart failure is a catabolic state and obese individuals may have more metabolic reserve and are thus better able to “survive.” Also, there is little disagreement that cachexia is associated with poorer prognosis in heart failure [51]. It has also been argued that obesity imposes “compulsory exercise” on the patient; if work is a product of distance and mass, then obese persons may be working harder than leaner counterparts because of the greater mass; considering exercise is recommended in heart failure, this may be a reason for the better prognosis in obese individuals [52].

*Strengths and Limitations.* The strengths of this review include the fact that it reviews only studies that use internationally recognized definitions of obesity and heart failure; also advantageous is the fact that this review includes only studies with single, rather than composite, endpoints. By narrowing down to heart failure specifically, this review synthesizes the available evidence for a particularly important clinical subpopulation and strengthens the knowledge on the existence of the obesity paradox for this specific condition. The review highlights the fact that the paradox exists for other measures of body fat composition other than BMI, namely, the WC and TSF.

This review has limitations; most of them are direct results of the quality of studies included in the review. Some used unclear reporting methods, especially for baseline characteristics and some had imbalanced cohorts [36, 43, 48]. All studies used indirect measurements of body composition to evaluate body fat mass; most studies report BMI as the weight measure; the validity of the BMI as a predictor of body fat related health outcomes continues to generate debate [41]. All studies measure weight after disease onset with its concomitant fluid retention and thus may not represent true “dry weight.”

## 10. Future Directions

As demonstrated by the current review, the obesity paradox is apparent in heart failure patients, with adverse associations only apparent at extremely high BMI levels (usually > 40). The significance of this will require a review of the prevalent practice of asking obese persons with heart failure to lose weight. However, any such recommendation regarding weight must be consequent upon more conclusive evidence on the mechanisms of the obesity paradox and being certain that this is not a result of collider bias [53]. It therefore follows that more conclusive research on the etiology of heart failure in obesity must be conducted; until such a time, clinical management of heart failure patients must be carefully calibrated to suit individual patient circumstances. What remains clear, however, is that the practice of asking heart failure patients to lose weight is not supported by empirical evidence.

## 11. Conclusions

There is now increasing consensus that obesity may be associated with a better prognosis in heart failure. There are, however, concerns that these associations seen may be a result of collider bias or an unassessed effect of physical fitness, irrespective of obesity category. More research should be directed at understanding the mechanisms of this protection offered by obesity; this will help in tailoring patient management for optimal outcomes in this population.

## Figures and Tables

**Table 1:** EPPHP component and global ratings tool for quantitative studies.

(A) Selection bias	Strong 1	Moderate 2	Weak 3

(B) Study design	Strong 1	Moderate 2	Weak 3

(C) Confounders	Strong 1	Moderate 2	Weak 3

(D) Blinding	Strong 1	Moderate 2	Weak 3

(E) Data collection method	Strong 1	Moderate 2	Weak 3

(F) Withdrawals and dropouts	Strong 1	Moderate 2	Weak 3

Global rating for paper is as follows:

(1) Strong (no weak ratings).

(2) Moderate (one weak rating).

(3) Weak (two or more weak ratings).

**Table 2:** Study characteristics and results.

Author	Country	Design	Sample size	Follow-up time	Median/mean age	Weight status measure	Self-report/measured	Outcome parameter	Results	Quality	Limitations
Zamora et al. (2013) [47]	Spain	Prospective cohort	504	6.1 years (IQR 2.2–7.8)	68 years (IQR 58–74)	BMI	Measured	Hazard ratio	Lower risk of death associated with obesity. Hazard ratios were 2.08 (1.16–3.75), 0.88 (0.54–1.43), and 0.49 (0.28–0.86) for underweight, overweight, and obese patients, respectively.	Strong	

Casas-Vara et al. (2012) [42]	Spain	Prospective cohort	244	984 days (2.7 years)	(83.2 ± 0.5)	BMI	Measured	Survival	Overall median survival was 984 days with better survival as BMI increased; log ranks were 5.26, 8.45, and 10.95 for underweight, overweight, and obese patients, respectively.	Moderate	Baseline characteristics of study population were not fully reported.

Lavie et al. (2013) [43]	USA	Prospective cohort	2066	5 years (25.0 ± 17.5 months)		BMI	Measured	5-year survival	Overall better prognosis in patients with higher BMI. On stratification on high FIT versus low FIT, obesity paradox persists in the high fit group but not in the low fit group.	Moderate	Primary objective was comparison between low fit versus high fit and not obese versus lean.

Gastelurrutia et al. (2011) [44]	Spain	Prospective cohort	979	44 months	65 ± 12 years	BMI	Measured	Hazard ratio	Lower risk of death associated with higher BMI when compared to normal BMI = 0.94, (0.91–0.97).	Strong	

Clark et al. (2011) [36]	USA	Retrospective cohort	344	2 years	53.3 ± 13.1 years	BMI, WC	Measured	2-year survival	Higher 2-year survival in high versus normal WC: 77.9% versus 64.3%, and high versus normal BMI: 89.8% versus 58.2%.	Moderate	Retrospective analysis of data of patients seen at a specialist center for heart transplant patients selected based on disease severity and prognosis and thus not likely to be representative of all heart failure patients.

Clark et al. (2012) [35]	USA	Retrospective cohort	2718	2 years	53.0 ± 12.4 years	BMI, WC	Measured	2-year survival	Higher 2-year survival with increasing BMI in both sexes. Men: high versus normal, BMI (63.2% versus 53.5%), WC (78.8% versus 63.1), women: BMI (67.1% versus 56.6%), WC, no difference.	Moderate	Not many women were included (~25%), bringing into question the power to detect differences in that subgroup.

Schwartzenberg et al. (2012) [48]	Israel	Prospective cohort	2323	15 months	71.6 ± 12.5 years	BMI	Not stated	Hazard ratio	Lower risk of death in persons with higher BMI. Normal versus higher BMI, though not statistically significant after adjustment. Hazard ratio of 0.79 (95% CI 0.59–1.05).	Moderate	Important baseline characteristics not shown.

Kapoor and Heidenreich (2010) [49]	USA	Retrospective cohort	1236	426 ± 461 days	71 ± 12 years	BMI	Measured	Hazard ratio	Lower risk of death as BMI increased until BMI > 45. Hazard ratios were 1.68 (95% CI, 1.04–2.69), 0.99 (95% CI, 0.71–1.36), 0.58 (95% CI, 0.35–0.97), 0.79 (95% CI, 0.44–1.4), and 1.38 (95% CI 0.74–2.6) for underweight, overweight, obese, and morbidly obese patients and BMI > 45 kg/m^2^, respectively. Thus, underweight and BMI > 45 kg/m^2^ were associated with increased risk of death.	Strong	

Curtis et al. (2005) [45]	USA	Clinical trial	7767	37 months	63.9 ± 10.9 years	BMI	Measured	Hazard ratio	Lower risk of death associated with increasing BMI. Using normal BMI as reference, hazard ratios were 1.21 (0.95–1.53), 0.88 (0.80–0.96), and 0.81 (0.72–0.92) for underweight, overweight, and obese patients, respectively.	Moderate	Results were primarily for a *Digitalis* clinical trial; thus, results were reanalyzed with a new hypothesis that was specified after study.

Zuchinali et al. (2013) [46]	Brazil	Prospective cohort	344	30 ± 8.2 months	59 ± 13	TSF	Measured	Hazard ratio	TSF > 20 mm was a strong predictor of all-cause mortality. Hazard ratio = 0.36 (0.13–0.97, *p* = 0.03). Results for other measures of body fat composition, BMI, WC, arm circumference (AC), Arm Muscle Circumference (AMC), and TSF, were not statistically significant in any direction.	Strong	

## References

[1] Popkin B. M., Doak C. M. (1998). The obesity epidemic is a worldwide phenomenon. *Nutrition Reviews*.

[2] World Health Organization Obesity and Overweight. http://www.who.int/mediacentre/factsheets/fs311/en/.

[3] Flegal K. M., Carroll M. D., Ogden C. L., Johnson C. L. (2002). Prevalence and trends in obesity among US adults, 1999-2000. *The Journal of the American Medical Association*.

[4] Manson J. E., Bassuk S. S. (2003). Obesity in the United States: a fresh look at its high toll. *The Journal of the American Medical Association*.

[5] Mattei J., Malik V., Wedick N. M. (2012). A symposium and workshop report from the Global Nutrition and Epidemiologic Transition Initiative: nutrition transition and the global burden of type 2 diabetes. *British Journal of Nutrition*.

[6] Calle E. E., Thun M. J., Petrelli J. M., Rodriguez C., Heath C. W. (1999). Body-mass index and mortality in a prospective cohort of U.S. adults. *The New England Journal of Medicine*.

[7] Diehr P., Bild D. E., Harris T. B., Duxbury A., Siscovick D., Rossi M. (1998). Body mass index and mortality in nonsmoking older adults: the cardiovascular health study. *American Journal of Public Health*.

[8] Peeters A., Barendregt J. J., Willekens F. (2003). Obesity in adulthood and its consequences for life expectancy: a life-table analysis. *Annals of Internal Medicine*.

[9] Kenchaiah S., Evans J. C., Levy D. (2002). Obesity and the risk of heart failure. *The New England Journal of Medicine*.

[10] Dunlap S. H., Sueta C. A., Tomasko L., Adams K. F. (1999). Association of body mass, gender and race with heart failure primarily due to hypertension. *Journal of the American College of Cardiology*.

[11] Katzmarzyk P. T., Janssen I., Ardern C. I. (2003). Physical inactivity, excess adiposity and premature mortality. *Obesity Reviews*.

[12] Lavie C. J., Milani R. V. (2003). Obesity and cardiovascular disease: the hippocrates paradox?. *Journal of the American College of Cardiology*.

[13] McAuley P. A., Blair S. N. (2011). Obesity paradoxes. *Journal of Sports Sciences*.

[14] Aursulesei V., Cozma A., Datcu M. D. (2009). Obesity paradox. *Revista Medico-Chirurgicala a Societatii de Medici si Naturalisti din Iasi*.

[15] Barry V. W., Baruth M., Beets M. W., Durstine J. L., Liu J., Blair S. N. (2014). Fitness vs. fatness on all-cause mortality: a meta-analysis. *Progress in Cardiovascular Diseases*.

[16] Flegal K. M., Kit B. K., Orpana H., Graubard B. I. (2013). Association of all-cause mortality with overweight and obesity using standard body mass index categories: a systematic review and meta-analysis. *The Journal of the American Medical Association*.

[17] Flegal K. M., Kalantar-Zadeh K. (2013). Overweight, mortality and survival. *Obesity*.

[18] Andreotti F., Rio T., Lavorgna A. (2009). Body mass index and cardiovascular events: the ‘obesity paradox’. *Recenti Progressi in Medicina*.

[19] Oreopoulos A., Padwal R., McAlister F. A. (2010). Association between obesity and health-related quality of life in patients with coronary artery disease. *International Journal of Obesity*.

[20] Zafrir B., Adir Y., Shehadeh W., Shteinberg M., Salman N., Amir O. (2013). The association between obesity, mortality and filling pressures in pulmonary hypertension patients; the ‘obesity paradox’. *Respiratory Medicine*.

[21] Stamou S. C., Nussbaum M., Stiegel R. M. (2011). Effect of body mass index on outcomes after cardiac surgery: is there an obesity paradox?. *Annals of Thoracic Surgery*.

[22] Badheka A. O., Rathod A., Kizilbash M. A. (2010). Influence of obesity on outcomes in atrial fibrillation: yet another obesity paradox. *American Journal of Medicine*.

[23] Lavie C. J., Milani R. V., Ventura H. O. (2011). Obesity and the ‘obesity paradox’ in cardiovascular diseases. *Clinical Pharmacology and Therapeutics*.

[24] Andreotti F., Rio T., Lavorgna A. (2009). Body fat and cardiovascular risk: understanding the obesity paradox. *European Heart Journal*.

[25] Blum A., Simsolo C., Sirchan R., Haiek S. (2011). ‘Obesity paradox’ in chronic obstructive pulmonary disease. *Israel Medical Association Journal*.

[26] Kunz K., Hannedouche T. (2009). Obesity in haemodialysis: the paradox. *Nephrologie et Therapeutique*.

[27] Park J., Ahmadi S.-F., Streja E. (2014). Obesity paradox in end-stage kidney disease patients. *Progress in Cardiovascular Diseases*.

[28] Tobias D. K., Pan A., Jackson C. L. (2014). Body-mass index and mortality among adults with incident type 2 diabetes. *The New England Journal of Medicine*.

[29] Kalantar-Zadeh K., Abbott K. C., Salahudeen A. K., Kilpatrick R. D., Horwich T. B. (2005). Survival advantages of obesity in dialysis patients. *The American Journal of Clinical Nutrition*.

[30] Artham S. M., Lavie C. J., Milani R. V., Ventura H. O. (2008). The obesity paradox: impact of obesity on the prevalence and prognosis of cardiovascular diseases. *Postgraduate Medicine*.

[31] Goel K., Lopez-Jimenez F., De Schutter A., Coutinho T., Lavie C. J. (2014). Obesity paradox in different populations: evidence and controversies. *Future Cardiology*.

[32] Horwich T. B., Fonarow G. C. (2002). The impact of obesity on survival in patients with heart failure. *Heart Failure Monitor*.

[33] Horwich T. B., Fonarow G. C., Hamilton M. A., MacLellan W. R., Woo M. A., Tillisch J. H. (2001). The relationship between obesity and mortality in patients with heart failure. *Journal of the American College of Cardiology*.

[34] Clark A. L., Fonarow G. C., Horwich T. B. (2014). Obesity and the obesity paradox in heart failure. *Progress in Cardiovascular Diseases*.

[35] Clark A. L., Chyu J., Horwich T. B. (2012). The obesity paradox in men versus women with systolic heart failure. *American Journal of Cardiology*.

[36] Clark A. L., Fonarow G. C., Horwich T. B. (2011). Waist circumference, body mass index, and survival in systolic heart failure: the obesity paradox revisited. *Journal of Cardiac Failure*.

[37] Kalantar-Zadeh K., Anker S. D., Coats A. J. S. (2005). Obesity paradox as a component of reverse epidemiology in heart failure. *Archives of Internal Medicine*.

[38] Lavie C. J., Osman A. F., Milani R. V., Mehra M. R. (2003). Body composition and prognosis in chronic systolic heart failure: the obesity paradox. *American Journal of Cardiology*.

[39] Lavie C. J., Ventura H. O. (2011). Weighing in on obesity and the obesity paradox in heart failure. *Journal of Cardiac Failure*.

[40] Thomas H. (2003). *Quality Assessment Tool for Quantitative Studies*.

[41] Strohle A., Worm N. (2014). Healthy obesity? Why the adiposity paradox is only seemingly paradox. *Medizinische Monatsschrift für Pharmazeuten*.

[42] Casas-Vara A., Santolaria F., Fernández-Bereciartúa A., González-Reimers E., García-Ochoa A., Martínez-Riera A. (2012). The obesity paradox in elderly patients with heart failure: analysis of nutritional status. *Nutrition*.

[43] Lavie C. J., Cahalin L. P., Chase P. (2013). Impact of cardiorespiratory fitness on the obesity paradox in patients with heart failure. *Mayo Clinic Proceedings*.

[44] Gastelurrutia P., Pascual-Figal D., Vazquez R. (2011). Obesity paradox and risk of sudden death in heart failure: results from the MUerte Subita en Insuficiencia cardiaca (MUSIC) study. *American Heart Journal*.

[45] Curtis J. P., Selter J. G., Wang Y. (2005). The obesity paradox: body mass index and outcomes in patients with heart failure. *Archives of Internal Medicine*.

[46] Zuchinali P., Souza G. C., Alves F. D. (2013). Triceps skinfold as a prognostic predictor in outpatient heart failure. *Arquivos Brasileiros de Cardiologia*.

[47] Zamora E., Lupón J., De Antonio M. (2013). The obesity paradox in heart failure: is etiology a key factor?. *International Journal of Cardiology*.

[48] Schwartzenberg S., Benderly M., Malnick S., George J., Goland S. (2012). The ‘obesity paradox’: does it persist among Israeli patients with decompensated heart failure? a subanalysis of the heart failure survey in Israel (HFSIS). *Journal of Cardiac Failure*.

[49] Kapoor J. R., Heidenreich P. A. (2010). Obesity and survival in patients with heart failure and preserved systolic function: a U-shaped relationship. *American Heart Journal*.

[50] Sharma A., Lavie C. J., Borer J. S. (2015). Meta-analysis of the relation of body mass index to all-cause and cardiovascular mortality and hospitalization in patients with chronic heart failure. *The American Journal of Cardiology*.

[51] Anker S. D., Negassa A., Coats A. J. S. (2003). Prognostic importance of weight loss in chronic heart failure and the effect of treatment with angiotensin-converting-enzyme inhibitors: an observational study. *The Lancet*.

[52] Anker S. D., von Haehling S. (2011). The obesity paradox in heart failure: accepting reality and making rational decisions. *Clinical Pharmacology and Therapeutics*.

[53] Sanni Ali M., Groenwold R. H. H., Pestman W. R. (2013). Time-dependent propensity score and collider-stratification bias: an example of beta2-agonist use and the risk of coronary heart disease. *European Journal of Epidemiology*.

